# Consensus core outcome rating for the Japanese neonatal pain guidelines

**DOI:** 10.3389/fped.2023.1174222

**Published:** 2023-06-07

**Authors:** Takeshi Arimitsu, Mio Ozawa, Kaori Gaughwin

**Affiliations:** ^1^ Department of Pediatrics, Keio University School of Medicine, Tokyo, Japan; ^2^ Division of Nursing Science, Graduate School of Biomedical and Health Sciences, Hiroshima University, Hiroshima, Japan; ^3^ Japanese Organization for NICU Families, Tokyo, Japan

**Keywords:** consensus, GRADE, guideline, neonates, NICU, outcome, pain, parents

## Abstract

**Introduction:**

The Japanese Neonatal Pain Guidelines Committee, led by the Japan Academy of Neonatal Nursing, uses the Grading of Recommendations, Assessment, Development, and Evaluation Working Group method to evaluate the quality of evidence and the strength of treatment recommendations. Ratings on the importance of outcomes related to neonatal pain have not been reported. This study aimed to reach a consensus on the importance of outcomes through a guideline panel composed of doctors, nurses, a nurse practitioner, a physical therapist, and families to ensure consistency in systematic reviews of neonatal pain and future revisions to the guidelines.

**Methods:**

A total of 26 professionals, including 21 medical personnel from clinical settings and academia and 5 parents from five family associations, participated in 3-stage eDelphi rounds.

**Results:**

The literature review and discussion identified 75 outcomes that were included in round one. The participants proposed three additional outcomes: 78 outcomes were scored in rounds two and three. Round three scores showed different stakeholder groups in terms of priority outcomes. Seventeen outcomes were included in the final core outcome and were considered critical for decision-making.

**Conclusion:**

Core outcomes of the development of neonatal pain guidelines in Japan were identified. The assessment process of importance from this study highlights the difference in the perspectives of medical providers and parents on neonatal pain, thus, involving parents in the assessment and as the spokesperson for the infant admitted to the neonatal intensive care unit is important for a more inclusive evaluation of pain prevention and management.

## 1. Introduction

The Committee for the Establishment and Dissemination of the Japanese Guidelines for Pain Prevention and Management in the NICU is the entity responsible for developing and disseminating the guidelines, which are jointly developed by five academic organizations (Japan Academy of Neonatal Nursing, Japan Society for Neonatal Health and Development, Japan Society of Perinatal and Neonatal Medicine, Japanese Society of Anesthesiologists, and Japanese Society of Pediatric Surgeons) and five family associations; the third edition of the guidelines is scheduled to be published in 2025. The revised version of the third edition uses the Grading of Recommendations Assessment, Development, and Evaluations (GRADE) methodology to assess the certainty of the evidence and strengthen the recommendations. A systematic review of guideline development is required to identify the most significant outcomes for newborns. In the GRADE methodology, a rating of 1–9 classifies the importance of outcomes, with 1–3 meaning “low importance in decision-making”, 4–6 meaning “important but not critical”, and 7–9 meaning “important for decision-making” (1). Although many guidelines for neonatal pain have been published in Japan and abroad (2), none have reported the process of assessing the importance of outcomes or their results. Although not limited to pain, there is a previous study in which various stakeholders, including families, participated and extracted the core outcomes common to neonatal research (3). This report showed that survival, sepsis, necrotizing enterocolitis, brain injury, retinopathy of prematurity, motor performance, cognitive ability, quality of life, adverse events, vision, hearing, and chronic lung disease were the most important factors. However, these outcomes are not specific to neonatal pain and are insufficient for a systematic review of outcomes in the pain guidelines. The characteristic neonatal pain outcomes include the pain score, duration, threshold, and the number of skin punctures (4). Nurses tend to rate the severity of neonatal pain more strongly than doctors (5, 6), and neonatal pain is also a strong stressor for their families (7). A previous report showed that the degree of importance of outcomes differed depending on the stakeholder, and it was assumed that the perception of neonatal pain differed among medical professionals and family members (3). Therefore, to build a consensus on the extraction of important outcomes for newborns, it is necessary to incorporate the perspectives of patients who are subjected to medical care, in addition to those of multidisciplinary experts. As a spokesperson for the newborn, the family represents the newborn's perspective. It is important to incorporate family perspectives when assessing neonatal pain outcomes. This study aimed to reach a consensus on the importance of outcomes through a guideline panel composed of doctors, nurses, co-medicals, and families to ensure consistency in systematic reviews of neonatal pain and future revisions to the guidelines.

## 2. Materials and methods

### 2.1. Delphi survey

In this study, we used the Delphi method, which is often used for consensus-building among expert groups. The Delphi method, developed by the RAND Research Institute in the 1950s, is “a method for eliciting and refining group judgments” (8, 9). This method is typically evaluated multiple times, and each member of the expert group responds anonymously. The researcher (facilitator) reports the statistics, such as the average and median of each session and the descriptions (e.g., reasons for the response), to the group members and the experts answer again based on the results. Three surveys are typically conducted (8), and the final results are considered consensus-building. This is one of the methods proposed in many clinical practice guideline preparation manuals to show unbiased consensus guideline building (1, 10). Other methods besides the Delphi method include the nominal group technique, but since the patient's parents tend to have strong opinions in face-to-face discussions, the Delphi method was selected because it facilitates the submission of one's own opinions anonymously until the end of the discussion. In this study, we used the eDelphi method, which was conducted electronically without paper questionnaires or feedback.

### 2.2. Participants: members of the guideline panel

The members of the Committee for the Establishment and Dissemination of the Japanese Guidelines for Pain Prevention and Management in the NICU comprised 27 people, including clinicians engaged in neonatal pain care, medical professionals engaged in education and research in neonatal care, and representatives of parent associations, including seven pediatricians, five clinical nurses, one nurse certified in neonatal intensive care, four nursing researchers with three having neonatal nursing experiences in NICUs, one nurse who was a child life specialist working in the NICU, two pediatric anesthesiologists, one pediatric surgeon, one neonatal nurse practitioner, one physical therapist, and five parents. The selection criteria for the members of this committee were defined as being neonatal health care providers with an interest in neonatal pain care and many years of experience working in NICUs or parent caring for a child who was actually subjected to neonatal pain management. All panel members declared any conflicts of interest before the survey began, and we checked for any possible conflicts of interest among panel members with respect to decision-making and voting. To prevent unforeseen conflicts of interest, job titles were completely withheld when the results of the first and second rounds of voting and free writing were fed back. We also believe that the fact that this was a completely online vote and there was no face-to-face exchange of opinions further reduced the possibility of conflicts of interest among the members. There was no daily face-to-face relationship among members. The facilitator for this study was the chairperson of this committee (MO), and the chairperson did not participate in the importance rating vote. Supplementary Table S1 shows the demographic information of the 26 participants in the outcome importance rating ballot. All committee members were informed in advance that the results would be made public, and anonymous responses were deemed as consent to participate in this activity.

### 2.3. Development of the outcomes list and consensus process

The committee held online meetings in June, September, and December 2021 and in March and June 2022 to discuss pain in newborns. A total of 75 outcomes were obtained. Outcomes were identified in the existing pain care guidelines. The 75 outcomes extracted were those identified in the revised second edition of the guidelines published in Japan in 2020 and in guidelines on neonatal pain published outside Japan. In addition, to make it easier for parents to answer the questions, an explanatory text was added for outcomes that were difficult to understand. In June 2022, 75 outcomes were imported into an online survey software platform, and the chairperson emailed the response URL to all committee members (*n* = 26). The survey was anonymous, did not collect information about respondents, could not directly verify respondents' identities outside their job type, and did not create identification links. To maximize the responses, the questionnaire was maintained as short as possible. Committee members participated in building a consensus on the three eDelphi surveys. In each round, we asked them to rank outcomes between 1 and 9 according to the GRADE guidelines. Each member made assessments in terms of the effectiveness of pain care and the importance of the outcome for the newborn. In the first round, participants suggested outcomes that were not identified in the review as important, and these outcomes were included in the second and third rounds. In addition, in the second and third rounds, outcomes with divergent ratings in the previous round (median of seven or higher but with a rating of three or less) were answered with a description of the reasons for the assessment. The consensus criterion for “critical outcomes” in the guidelines was that the median of all respondents in the third round of evaluations was 7 or more but the median of those providing no response was 3 or less. After each round, the committee chairperson facilitated the study, compiled the scoring results, and produced a table showing the mean, median, minimum, and maximum values of each outcome. A list of scoring reasons was prepared for the outcome items with divergent evaluations. Before scoring in the second and third rounds, the study participants reviewed statistics such as the mean of each outcome and descriptions of the reasons for scoring. A list of reasons for grading was presented with hidden attributes.

### 2.4. Ethical considerations

The publication of the results of the eDelphi method conducted on the members of this panel was approved by the Hiroshima University Epidemiological Research Ethics Review Committee (license number: E2022-0119).

## 3. Results

### 3.1. Final core outcome set

**Figure 1:**
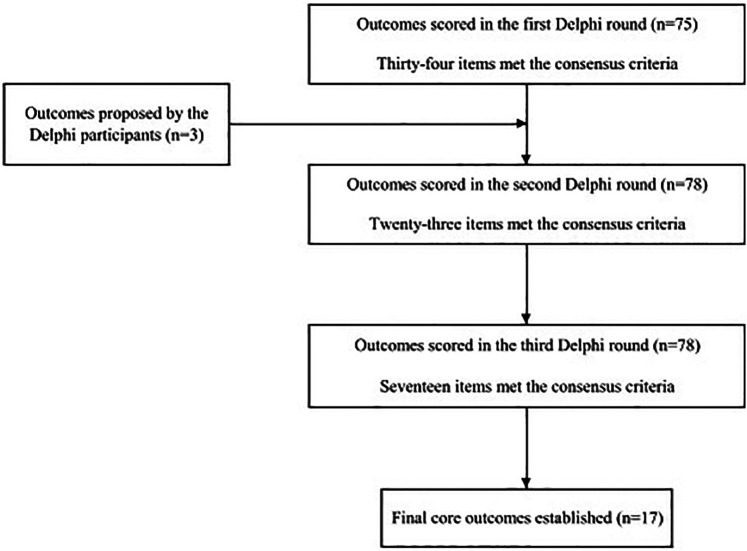
Flowchart of the identification and selection of outcomes.

Figure 1 shows the selection process for the outcomes that met the agreed criteria in the first, second, and third material assessments. Participants were provided with feedback on the results of the immediately preceding assessment of each outcome (mean, median, frequency, bar graph showing frequency distribution, and percentage) prior to the second and third rounds of assessment. Non-respondents from the previous round were also invited to each round 2 and 3. Seventeen outcomes met the predefined consensus criteria (Table 1). The descriptive analysis for each eDelphi round of the final core outcomes is shown in Supplementary Table S2. In this study, the consensus criterion for the importance of an outcome to be classified as “important for decision-making” in all rounds was “a median value of 7 or more and no rating below 3” (8).

**Table 1:** Final consensus outcomes (*n* = 17) from the three round eDelphi assessment of 78 items extracted from the Japanese neonatal pain guidelines.

Outcomes
Pain intensity
Duration of pain
Vital signs
Apnea
Multiple physiological indicators
Neurodevelopmental outcomes
Safe implementation of procedures
Saturation
Motor development
Bonding between parents and neonates
Bradycardia
Staff awareness of pain
Development index
Intelligence quotient
Developmental disorder
Pain threshold
Family Anxiety

The consensus criterion for the importance of an outcome to be classified as “critical outcome: important for decision-making” in all rounds was “a median value of 7 or more and no rating below 3”. Thirty-four outcomes met the criteria in the first round, twenty-three outcomes met the criteria in the second round. In the final round, seventeen outcomes met the criteria.

### 3.2. First round

The importance of 75 outcomes was assessed between June 4 and June 9, 2022, and responses were obtained from 23 individuals (the attrition percentage was 23/26, 88.5%): 11 nurses and therapists, 8 doctors, and 4 parents. Thirty-four outcomes met the consensus criteria for critical outcomes (median ≥7 but no responses ≤3). There were 22 items with a median score of ≥7 and a rating of ≤3, specifically “safe implementation of treatment”, “facial pallor”, “postoperative infection”, “wound inflammation”, “cry duration”, “pain threshold”, and “family anxiety”. Nineteen items were classified as non-critical but important outcomes (median, 4–6), and none were classified as less important (median, ≤3). In addition, three new parameters were proposed: height, head circumference, and physical development.

### 3.3. Second round

The importance of 78 outcomes was assessed between June 10 and June 20, 2022, and responses were obtained from 20 individuals (the attrition percentage was 20/26, 76.9%): 11 nurses and therapists, 5 doctors, and 4 parents. Twenty-three outcomes met the agreed criteria for consensus outcomes (median ≥7 and no responses ≤3), with “safe implementation of treatment”, “time spent crying”, “state”, and “pain threshold” being newly classified as critical outcomes. The number of items with divided ratings (median of ≥7 and ≤3) increased to 28. In the first study, the consensus criteria were met. However, in the second study, the outcomes that resulted in divergent assessments (median ≥7 and a rating of ≤3) included death, shock, complications in preterm infants, physical invasion due to surgery, neuropathy due to anesthesia procedures, number of painful procedures, and number of skin punctures. Non-critical but important outcomes (median 4–6) increased to 27 items, whereas less important outcomes (median ≤3) remained at 0.

### 3.4. Third round

The importance of 78 outcomes was assessed from June 21 to June 30, 2022, and responses were obtained from 23 individuals (the attrition percentage was 23/26, 88.5%), including 11 nurses and therapists, 8 doctors, and 4 parents. Seventeen outcomes met the consensus criteria for a critical outcome (median ≥7 but no responses ≤3), and “developmental disability” was newly classified as a critical criterion. The outcomes that met the consensus criteria in the second study but resulted in divergent assessments in the third study (median ≥7 and a rating of ≤3) were “crying,” “heart rate,” “blood pressure” and “state”. The number of non-critical but important outcomes (median, 4–6) increased to 37, whereas non-critical outcomes (median, ≤3) remained at 0. A descriptive analysis of all outcomes (78 items) is shown in Supplementary Table S3. Prior studies have recommended 2–3 rounds, but there is no scientific basis for determining the optimal number of rounds (11). Since the most commonly cited method is three rounds, as described in the RAND Institute's manual, we adopted three rounds in this study as well.

Table 2 shows the top ten outcomes for each stakeholder in the third round. Supplementary Table S3 shows a descriptive analysis of each outcome. The common outcomes for each stakeholder were pain duration, pain score, and vital signs. Apnea, oxygen saturation, and other complications in preterm infants were common only among families, nurses, and therapists. Parent members and doctors had one thing in common: death and shock. Outcomes cited only by parent members included facial pallor, heart rate, blood pressure, pneumonia, and pneumothorax, with clinical symptoms that visually induced parental anxiety. Outcomes cited only by nurses, nurse practitioner and pysical therapist included safe administration of procedures, oxygen saturation, developmental impairment, and wound inflammation. Outcomes cited only by physicians included motor development, developmental index, bradycardia, gastrointestinal perforation after surgery, and parents-neonatal bonding.

**Table 2:** Top 10 outcomes from each stakeholder group in the third round.

Parents (*n* = 4)	Nurses, NP, and PT (*n* = 11)	Doctors (*n* = 8)
Death	Duration of pain	Pain score
Apnea	Pain score	Death
Duration of pain	Vital signs	Duration of pain
Saturation	Apnea	Neurodevelopmental outcomes
Shock condition	Multiple physiological indicators	Shock condition
Pain score	Complications of preterm birth	Multiple physiological indicators
Vital signs	Neurodevelopmental outcomes	Motor development
Pallor of the face	Safe implementation of procedures	Development index
Heart rate	Saturation	Intelligence quotient
Blood pressure	Developmental disorder	Vital signs
Complications of preterm birth	Wound inflammation	Bradycardia
Pneumonia Pneumothorax	Staff awareness of pain	Perforation of the gastrointestinal tract after surgery
		Bonding between parents and neonates

The outcomes were ranked based on the mean scores for each item. As there are several outcomes with the same mean value, more than 10 outcomes are listed in each group. NP, nurse practitioner; PT, physical therapist.

## 4. Discussion

In this study, we evaluated the importance of outcomes in the neonatal pain guidelines from the perspectives of healthcare providers involved in NICU care and families of newborns. Ultimately, 17 outcomes were extracted as “critical outcomes” (Table 1). Although several studies have attempted to extract and assess pain outcomes in children (11, 12), newborns were excluded from the studies. To our knowledge, the core outcomes of neonatal pain extracted in this study are the first. The results of this study suggest that “pain score” and “pain duration”, which indicate the degree of pain, are the most important pain outcomes in newborns as well as in children aged 3 years and older (12). Outcomes indicating pain symptoms and adverse events, such as “vital signs”, “apnea”, “oxygen saturation”, “bradycardia”, and “safe procedures” have been shown to be important. Many of these outcomes overlapped with those identified in previous studies (3) as important evaluation indicators for research in the neonatal area, regardless of pain.

The final core outcomes (Table 1) included all outcomes that were common to each stakeholder and all outcomes that were common only to the parents, nurses, and therapists in the ranking of the average of the top ten outcomes from each stakeholder (Table 2). This result indicates that the core outcomes included indicators that were commonly considered important by each stakeholder and those that were considered important by a specific stakeholder. Although the purpose of the outcome assessment was clearly stated to the evaluators well in advance and explained to them, differences in experience and position may have influenced the divergence. For example, death was selected as a top choice by parents and physicians, but not by nurses, NPs, or PTs, and did not appear on the final list of core outcomes (Table 1). Supplementary Table S3 indicates that the mean score for “death” was almost identical for physicians and nurses, but was 1.5–2.0 points higher for parents. In addition, only the physician ratings included responses with an importance rating of 3. This indicates that the viewpoints and degree of importance varied by position, and it is possible that the findings would have changed if the composition of the panel (parents, ratio of physicians to nurses, etc.) or the weight of each group's ratings had changed. The composition of the panel in this study reflected a diverse range of ages, genders, and occupations, and the consensus, at least from the various perspectives in this study, was that death was not selected as a pain outcome. The results of this study were also consistent with previous research on pain outcomes for children (11, 12), as death was not selected as the final outcome in the study. The indicators considered important by each stakeholder were not completely consistent, indicating that consensus building by various stakeholders was necessary for the outcome evaluation of guidelines and that this could be achieved using the Delphi method. Especially in neonatal care, the parents of a newborn represent the interests of their baby who is being treated and is also directly affected by the outcomes of neonatal care. Therefore, it is important to incorporate parents' perspectives into neonatal care.

Table 2 and Supplementary Table S3 show that the newborn's parents, doctors, and nurses have clearly different perspectives and priorities This may be because physicians are primarily responsible for making treatment decisions, whereas nurses provide bedside care for the newborn and spend more time with the parents. In contrast, it is interesting to note that among the highest scoring of the top ten outcomes by the stakeholder group (Table 2), five of the 13 outcomes were mentioned by families only, and only six outcomes, representing approximately half of the outcomes, were shared by families, nurses, and therapists.

Outcomes mentioned only by parent members included pallor, heart rate, blood pressure, pneumonia, and pneumothorax, which were associated with anxiety when the parents noticed these clinical symptoms in their infants (Table 2). This may be because parents are particularly concerned about the clinical symptoms of the neonate in front of them and their impression of the clinical symptoms of the neonate during hospitalization. This may be related to the fact that families of neonates admitted to the NICU have a high emotional burden and are likely to be anxious and worried even after their child is discharged. In light of these findings, the results suggest the need for bedside psychological support for families of newborns admitted to the NICU. However, the outcomes mentioned only by nurses and physicians included developmental disabilities, motor development, developmental index, and bond formation between the parents and newborns, which were mostly related to long-term prognosis and other issues after discharge from the NICU. This may be due to families' lack of knowledge about the impact of NICU-induced neonatal pain on neurological development and parent relationships after the neonate is discharged from the NICU (e.g., the impact of neonatal pain on prognosis and the need for education). These results suggest the need for enhanced opportunities to educate families about the importance of pain care for newborns in the NICU. The final core outcomes included a good balance of opinions among parent members, nurses, therapists, and physicians (Table 1). This finding suggests that the outcome evaluation was appropriately conducted and validated. The outcomes of this study were broadly divided into two categories: those related to methods for directly measuring pain sensation and those related to the effects of pain on the infants themselves and those around them who experience it. While previous studies on neonatal pain have generally focused on the former, this study addressed both. This is because systematic efforts to prevent and alleviate neonatal pain, including education of health care providers, pain care protocols, and documentation, are important. Outcomes related to patient parent members were also included because of their participation as evaluators.

There were some limitations in this study. One limitation is that evaluation of importance of pain outcomes was based on the level of neonatal care and nursing in Japan. The cost of neonatal care in Japan is financed by the universal health insurance system, and the financial burden on families is minimal, even for advanced medical care. Therefore, cost-related outcomes, which were extracted in previous studies (11, 12), were not considered highly important in this study. In addition, under Japan's Maternal Protection Law, fetuses born at 22 weeks or more are eligible for life-saving care, and healthcare providers are highly interested in post-discharge outcomes related to the development of very preterm infants. Therefore, it is possible that developmental outcomes were rated more highly in the importance assessment in this study and that their importance may differ in countries where neonatal care differs from that in Japan. In addition, the failure to maintain a response rate of approximately 90% for all rounds is a limitation of this study, even though active efforts were put in to reduce the number of dropouts, with the deadline for voting made known to the entire group, and voting reminders were sent out frequently.

## 5. Conclusion

The eDelphi method, which included the patient's parents as evaluators, was used to assess the importance of neonatal pain guideline outcomes, and 17 “critical outcomes” were identified. The assessment process of importance showed that the perspectives of medical providers and families on neonatal pain differed; further, the importance of including parents in the assessment as the spokesperson for the neonate admitted to the NICU is prominent.

## Data Availability

Raw data were generated at Hiroshima University, Japan. Data supporting the findings of this study are available from the corresponding author [MO] upon request.
