# Supplementary material for: Consensus core outcome rating for the Japanese neonatal pain guidelines
Source: Front Pediatr. 2023 Jun 7;11:1174222. doi: 10.3389/fped.2023.1174222 (PMC10282745; doi:10.3389/fped.2023.1174222)
Supplement: Supplementary file 1 [file Table1.docx]

| Supplementary Table 1. Demographic information of participants (N=26) | | | | | |
| --- | --- | --- | --- | --- | --- |
|  | Age | | | |  |
|  | 30s | 40s | 50s | 60s |  |
| Nurses and therapist (n=11) | 1(1) | 3(2) | 3(1) | 3(2) |  |
| Physicians (n=10) | 3(0) | 6(0) | 2(1) | 0 |  |
| Parents (n=5) | 1(1) | 3(0) | 1(0) | 0 |  |
| Total | 5(1) | 12(2) | 6(2) | 3(2) |  |
| Note: Numbers in parentheses indicate the number of males. | | | |  | |
