# Supplementary material for: Consensus core outcome rating for the Japanese neonatal pain guidelines
Source: Front Pediatr. 2023 Jun 7;11:1174222. doi: 10.3389/fped.2023.1174222 (PMC10282745; doi:10.3389/fped.2023.1174222)
Supplement: Supplementary file 2 [file Table2.docx]

Supplementary Table 2. The descriptive analyses for each eDelphi round for the final core outcomes.

| Outcomes | Round | Mean | SD | Min | Max | Median | 25^th^ percentile | 75^th^ percentile |
| --- | --- | --- | --- | --- | --- | --- | --- | --- |
| Pain intensity | 1 | 7.9 | 1.1 | 5 | 9 | 8 | 7 | 9 |
|  | 2 | 8.1 | 1.1 | 6 | 9 | 8 | 7 | 9 |
|  | 3 | 7.9 | 1.1 | 5 | 9 | 8 | 7 | 9 |
| Duration of pain | 1 | 8.0 | 1.2 | 5 | 9 | 9 | 7 | 9 |
|  | 2 | 8.1 | 1.1 | 6 | 9 | 8.5 | 7 | 9 |
|  | 3 | 7.8 | 1.3 | 4 | 9 | 8 | 7 | 9 |
| Vital signs | 1 | 7.6 | 1.5 | 5 | 9 | 7 | 6 | 9 |
|  | 2 | 7.7 | 1.2 | 6 | 9 | 7.5 | 7 | 9 |
|  | 3 | 7.2 | 1.5 | 4 | 9 | 7 | 6 | 8 |
| Apnea | 1 | 8.1 | 1.1 | 5 | 9 | 8 | 7 | 9 |
|  | 2 | 7.8 | 1.2 | 5 | 9 | 8 | 7 | 9 |
|  | 3 | 7.2 | 1.3 | 4 | 9 | 7 | 6 | 8 |
| Multiple physiological indicators | 1 | 7.3 | 1.5 | 5 | 9 | 8 | 6 | 9 |
|  | 2 | 7.7 | 1.3 | 5 | 9 | 8 | 6.25 | 9 |
|  | 3 | 7.2 | 1.4 | 4 | 9 | 7 | 6 | 8 |
| Neurodevelopmental outcomes | 1 | 8.0 | 1.0 | 6 | 9 | 8 | 7 | 9 |
|  | 2 | 7.3 | 1.4 | 4 | 9 | 7 | 6.25 | 8.25 |
|  | 3 | 7.2 | 1.1 | 5 | 9 | 7 | 6 | 8 |
| Safe implementation of procedures | 1 | 7.7 | 1.6 | 3 | 9 | 8 | 7 | 9 |
|  | 2 | 7.5 | 1.4 | 4 | 9 | 8 | 6.25 | 8.75 |
|  | 3 | 7.0 | 1.1 | 4 | 9 | 7 | 6 | 8 |
| Saturation | 1 | 7.4 | 1.3 | 5 | 9 | 7 | 6 | 9 |
|  | 2 | 7.4 | 1.2 | 5 | 9 | 7 | 7 | 8.75 |
|  | 3 | 6.9 | 1.3 | 4 | 9 | 7 | 6 | 8 |
| Motor development | 1 | 7.3 | 1.1 | 5 | 9 | 7 | 7 | 8 |
|  | 2 | 7.0 | 1.3 | 4 | 9 | 7 | 6.25 | 8 |
|  | 3 | 6.9 | 1.1 | 5 | 9 | 7 | 6 | 8 |
| Bonding between parents and neonates | 1 | 7.6 | 1.4 | 4 | 9 | 8 | 7 | 9 |
|  | 2 | 7.4 | 1.4 | 4 | 9 | 7 | 6.25 | 8.75 |
|  | 3 | 6.9 | 1.2 | 4 | 9 | 7 | 6 | 8 |
| Bradycardia | 1 | 7.5 | 1.2 | 5 | 9 | 8 | 7 | 9 |
|  | 2 | 7.3 | 1.2 | 5 | 9 | 7 | 7 | 8 |
|  | 3 | 6.8 | 1.2 | 4 | 9 | 7 | 6 | 8 |
| Staff awareness of pain | 1 | 7.3 | 1.7 | 4 | 9 | 7 | 6 | 9 |
|  | 2 | 7.2 | 1.5 | 5 | 9 | 7 | 6 | 8.75 |
|  | 3 | 6.8 | 1.3 | 4 | 9 | 7 | 6 | 8 |
| Development index | 1 | 7.3 | 1.1 | 5 | 9 | 7 | 7 | 8 |
|  | 2 | 7.2 | 1.2 | 4 | 9 | 7 | 7 | 8 |
|  | 3 | 6.7 | 1.1 | 4 | 9 | 7 | 6 | 7 |
| Intelligence quotient | 1 | 7.1 | 1.0 | 5 | 9 | 7 | 6 | 8 |
|  | 2 | 7.0 | 1.4 | 4 | 9 | 7 | 6.25 | 8 |
|  | 3 | 6.7 | 1.0 | 4 | 9 | 7 | 6 | 7 |
| Developmental disorder | 1 | 7.3 | 1.1 | 5 | 9 | 7 | 7 | 8 |
|  | 2 | 6.8 | 1.5 | 3 | 9 | 7 | 6.25 | 7.75 |
|  | 3 | 6.7 | 1.3 | 4 | 9 | 7 | 6 | 8 |
| Pain threshold | 1 | 7.0 | 1.7 | 3 | 9 | 7 | 6 | 8 |
|  | 2 | 7.0 | 1.2 | 5 | 9 | 7 | 6 | 8 |
|  | 3 | 6.7 | 1.2 | 4 | 9 | 7 | 6 | 8 |
| Family Anxiety | 1 | 7.0 | 1.6 | 3 | 9 | 7 | 6 | 9 |
|  | 2 | 6.9 | 1.3 | 4 | 9 | 7 | 6 | 8 |
|  | 3 | 6.7 | 1.2 | 5 | 9 | 7 | 6 | 8 |
