# Supplementary material for: Consensus core outcome rating for the Japanese neonatal pain guidelines
Source: Front Pediatr. 2023 Jun 7;11:1174222. doi: 10.3389/fped.2023.1174222 (PMC10282745; doi:10.3389/fped.2023.1174222)
Supplement: Supplementary file 3 [file Table3.docx]

Supplementary Table 3. Descriptive analysis of all outcomes for each stakeholder at the final round.

| Outcomes | Stakeholder | Mean | SD | Min | Max | Median | 25^th^ percentile | 75^th^ percentile |
| --- | --- | --- | --- | --- | --- | --- | --- | --- |
| Pain intensity | Nurses | 8.0 | 1.2 | 5 | 9 | 8 | 8 | 9 |
|  | Physician | 7.9 | 1.1 | 6 | 9 | 8 | 7 | 9 |
|  | Parents | 7.8 | 1.5 | 6 | 9 | 8 | 6.3 | 9 |
| Duration of pain | Nurses | 8.9 | 1.2 | 5 | 9 | 8 | 8 | 9 |
|  | Physician | 7.3 | 1.6 | 4 | 9 | 7 | 7 | 8.8 |
|  | Parents | 8.0 | 1.2 | 7 | 9 | 8 | 7 | 9 |
| Vital signs | Nurses | 7.5 | 1.5 | 4 | 9 | 8 | 7 | 9 |
|  | Physician | 6.6 | 1.5 | 4 | 8 | 7 | 5.3 | 8 |
|  | Parents | 7.8 | 1.5 | 6 | 9 | 8 | 6.3 | 9 |
| Apnea | Nurses | 7.4 | 1.1 | 5 | 9 | 8 | 7 | 8 |
|  | Physician | 6.4 | 1.2 | 4 | 8 | 6.5 | 6 | 7 |
|  | Parents | 8.5 | 1.0 | 7 | 9 | 9 | 7.5 | 9 |
| Multiple  physiological indicators | Nurses | 7.4 | 1.4 | 4 | 9 | 8 | 7 | 8 |
|  | Physician | 7.0 | 1.7 | 4 | 9 | 7 | 6 | 8.8 |
|  | Parents | 7.3 | 1.3 | 6 | 9 | 7 | 6.3 | 8.5 |
| Neurodevelopmental outcomes | Nurses | 7.4 | 1.0 | 6 | 9 | 7 | 7 | 8 |
|  | Physician | 7.1 | 1.5 | 5 | 9 | 7 | 6 | 8.8 |
|  | Parents | 6.8 | 0.5 | 6 | 7 | 7 | 6.3 | 7 |
| Safe implementation  of procedures | Nurses | 7.3 | 0.9 | 6 | 9 | 7 | 7 | 8 |
|  | Physician | 6.5 | 1.3 | 4 | 8 | 6.5 | 6 | 7.8 |
|  | Parents | 7.0 | 1.4 | 6 | 9 | 6.5 | 6 | 8.5 |
| Saturation | Nurses | 7.2 | 0.8 | 6 | 8 | 7 | 7 | 8 |
|  | Physician | 5.9 | 1.5 | 4 | 8 | 6 | 4.3 | 7 |
|  | Parents | 8 | 1.2 | 7 | 9 | 8 | 7 | 9 |
| Motor development | Nurses | 6.8 | 1.3 | 5 | 9 | 7 | 6 | 8 |
|  | Physician | 6.9 | 1.1 | 5 | 9 | 7 | 6.3 | 7 |
|  | Parents | 7 | 1.2 | 6 | 8 | 7 | 6 | 8 |
| Bonding between  parents and neonates | Nurses | 6.6 | 1.2 | 5 | 9 | 7 | 6 | 8 |
|  | Physician | 6.6 | 1.5 | 4 | 9 | 6.5 | 6 | 7.8 |
|  | Parents | 7.3 | 1.3 | 6 | 9 | 7 | 6.3 | 8.5 |
| Bradycardia | Nurses | 6.8 | 1.3 | 4 | 8 | 7 | 6 | 8 |
|  | Physician | 6.6 | 0.9 | 5 | 8 | 7 | 6 | 7 |
|  | Parents | 7.3 | 1.5 | 6 | 9 | 7 | 6 | 8.8 |
| Staff awareness of pain | Nurses | 7.1 | 1.2 | 5 | 9 | 7 | 7 | 8 |
|  | Physician | 6.3 | 1.6 | 4 | 9 | 6 | 5.3 | 7.5 |
|  | Parents | 7.3 | 1.3 | 6 | 9 | 7 | 6.3 | 8.5 |
| Development index | Nurses | 6.5 | 0.8 | 5 | 8 | 7 | 6 | 7 |
|  | Physician | 6.8 | 1.4 | 4 | 9 | 7 | 6.3 | 7 |
|  | Parents | 7.0 | 1.4 | 6 | 9 | 6.5 | 6 | 8.5 |
| Intelligence quotient | Nurses | 6.6 | 0.9 | 5 | 8 | 7 | 6 | 7 |
|  | Physician | 6.8 | 1.4 | 4 | 9 | 7 | 6.3 | 7 |
|  | Parents | 6.8 | 1.0 | 6 | 8 | 6.5 | 6 | 7.8 |
| Developmental disorder | Nurses | 7.0 | 1.0 | 6 | 9 | 7 | 6 | 8 |
|  | Physician | 6.4 | 1.8 | 4 | 9 | 7 | 4.3 | 7.8 |
|  | Parents | 6.3 | 0.5 | 6 | 7 | 6 | 6 | 6.8 |
| Pain threshold | Nurses | 6.6 | 1.2 | 5 | 8 | 7 | 5 | 8 |
|  | Physician | 6.5 | 1.2 | 4 | 8 | 7 | 6 | 7 |
|  | Parents | 7 | 1.8 | 5 | 9 | 7 | 5.3 | 8.8 |
| Family Anxiety | Nurses | 6.8 | 1.1 | 5 | 9 | 7 | 6 | 7 |
|  | Physician | 6.4 | 1.5 | 5 | 9 | 6 | 5 | 7.8 |
|  | Parents | 7 | 1.2 | 6 | 8 | 7 | 6 | 8 |
| Death | Nurses | 7.0 | 1.7 | 5 | 9 | 6 | 6 | 9 |
|  | Physician | 7.5 | 2.3 | 3 | 9 | 9 | 5.5 | 9 |
|  | Parents | 9.0 | 0 | 9 | 9 | 9 | 9 | 9 |
| Shock condition | Nurses | 6.9 | 1.8 | 3 | 9 | 7 | 6 | 8 |
|  | Physician | 7.0 | 2.1 | 4 | 9 | 7.5 | 5 | 9 |
|  | Parents | 8.0 | 1.2 | 7 | 9 | 8 | 7 | 9 |
| Complications of preterm birth | Nurses | 7.4 | 1.2 | 5 | 9 | 7 | 7 | 8 |
|  | Physician | 6.5 | 1.8 | 3 | 8 | 7 | 5.3 | 8 |
|  | Parents | 7.8 | 1.0 | 7 | 9 | 7.5 | 7 | 8.8 |
| Blood pressure | Nurses | 6.9 | 1.5 | 3 | 9 | 7 | 7 | 8 |
|  | Physician | 6.3 | 1.3 | 4 | 8 | 6 | 6 | 7.5 |
|  | Parents | 7.8 | 1.5 | 6 | 9 | 8 | 6.3 | 9 |
| Heart Rate | Nurses | 6.7 | 1.6 | 3 | 9 | 7 | 6 | 8 |
|  | Physician | 6.4 | 1.6 | 4 | 9 | 6 | 5.3 | 7.8 |
|  | Parents | 7.8 | 1.5 | 6 | 9 | 8 | 6.3 | 9 |
| Perforation of the  gastrointestinal tract after  surgery | Nurses | 6.6 | 1.7 | 2 | 8 | 7 | 6 | 8 |
|  | Physician | 6.6 | 1.8 | 3 | 9 | 7 | 5.5 | 7.8 |
|  | Parents | 7.5 | 1.7 | 6 | 9 | 7.5 | 6 | 9 |
| Pallor of the face | Nurses | 6.8 | 1.8 | 3 | 9 | 7 | 3.4 | 9 |
|  | Physician | 6.1 | 1.2 | 4 | 8 | 6 | 5.3 | 7 |
|  | Parents | 7.8 | 1.5 | 6 | 9 | 8 | 6.3 | 9 |
| Number of painful  procedures | Nurses | 6.9 | 1.6 | 3 | 9 | 7 | 6 | 8 |
|  | Physician | 5.8 | 1.3 | 4 | 7 | 6 | 4.3 | 7 |
|  | Parents | 7.5 | 1.7 | 6 | 9 | 7.5 | 6 | 9 |
| Number of skin punctures | Nurses | 7.0 | 1.5 | 3 | 9 | 7 | 7 | 8 |
|  | Physician | 5.6 | 1.2 | 4 | 7 | 6 | 4.3 | 6.8 |
|  | Parents | 7.3 | 1.5 | 6 | 9 | 7 | 6 | 8.9 |
| Creation of procedure manuals that take  into account pain | Nurses | 7.0 | 0.8 | 5 | 8 | 7 | 7 | 7 |
|  | Physician | 6.0 | 2.0 | 3 | 9 | 6 | 4.3 | 7.8 |
|  | Parents | 6.3 | 1.5 | 5 | 8 | 6 | 5 | 7.8 |
| Cry duration | Nurses | 6.9 | 1.1 | 5 | 9 | 7 | 6 | 8 |
|  | Physician | 5.5 | 1.4 | 3 | 7 | 6 | 4.3 | 6.8 |
|  | Parents | 7.5 | 1.3 | 6 | 9 | 7.5 | 6.3 | 8.8 |
| Physical invasion due to  surgery | Nurses | 7.0 | 1.3 | 5 | 9 | 7 | 6 | 8 |
|  | Physician | 5.6 | 1.7 | 3 | 7 | 6 | 3 | 7 |
|  | Parents | 7.0 | 2.5 | 4 | 9 | 7.5 | 4.5 | 9 |
| Neuropathy due to anesthesia  procedures | Nurses | 6.5 | 0.8 | 5 | 8 | 7 | 6 | 7 |
|  | Physician | 6.5 | 1.4 | 3 | 7 | 7 | 3 | 7 |
|  | Parents | 6.5 | 1.0 | 6 | 8 | 6 | 6 | 7.5 |
| Success rate of painful  procedures | Nurses | 6.9 | 1.6 | 3 | 9 | 7 | 6 | 8 |
|  | Physician | 5.6 | 1.2 | 4 | 7 | 6 | 4.3 | 6.8 |
|  | Parents | 7.0 | 1.8 | 5 | 9 | 7 | 5.3 | 8.8 |
| Development equivalent to  weeks of gestation | Nurses | 6.5 | 0.9 | 5 | 8 | 6 | 6 | 7 |
|  | Physician | 6.4 | 1.6 | 4 | 9 | 6 | 5.3 | 7.8 |
|  | Parents | 6.8 | 1.7 | 5 | 9 | 6.5 | 5.3 | 8.5 |
| Family satisfaction | Nurses | 6.5 | 0.9 | 5 | 8 | 7 | 6 | 7 |
|  | Physician | 6.5 | 1.4 | 5 | 9 | 6 | 5.3 | 7.8 |
|  | Parents | 6.5 | 1.7 | 5 | 9 | 6 | 5.3 | 8.3 |
| Opportunities to learn neonatal pain and pain management | Nurses | 6.8 | 0.8 | 5 | 8 | 7 | 7 | 7 |
|  | Physician | 6.0 | 2.1 | 3 | 9 | 6 | 4.3 | 8.3 |
|  | Parents | 6.3 | 1.5 | 5 | 8 | 6 | 5 | 7.8 |
| Family participation | Nurses | 7.0 | 1.1 | 5 | 9 | 7 | 6 | 8 |
|  | Physician | 5.8 | 1.4 | 3 | 7 | 6 | 5 | 7 |
|  | Parents | 6.3 | 0.5 | 6 | 7 | 6 | 6 | 6.8 |
| Family psychological burden | Nurses | 6.6 | 1.3 | 4 | 9 | 7 | 6 | 7 |
|  | Physician | 6.0 | 1.5 | 4 | 9 | 6 | 5 | 6.8 |
|  | Parents | 6.8 | 1.5 | 5 | 8 | 7 | 5.3 | 8 |
| State | Nurses | 6.7 | 1.3 | 4 | 9 | 7 | 6 | 7 |
|  | Physician | 5.6 | 1.5 | 3 | 7 | 6 | 4.3 | 7 |
|  | Parents | 7.0 | 1.4 | 6 | 9 | 6.5 | 6 | 8.5 |
| Unplanned extubation | Nurses | 7.0 | 1.7 | 3 | 9 | 8 | 6 | 8 |
|  | Physician | 5.0 | 2.0 | 2 | 7 | 6 | 3 | 6.8 |
|  | Parents | 7.5 | 1.9 | 5 | 9 | 8 | 5.5 | 9 |
| Duration of painful procedures | Nurses | 6.7 | 1.5 | 3 | 8 | 7 | 6 | 8 |
|  | Physician | 5.5 | 1.4 | 4 | 7 | 5.5 | 4 | 7 |
|  | Parents | 7.3 | 2.1 | 5 | 9 | 7.5 | 5.3 | 9 |
| Family confident | Nurses | 6.8 | 1.4 | 4 | 9 | 7 | 6 | 8 |
|  | Physician | 5.9 | 1.9 | 3 | 9 | 6 | 4.3 | 7 |
|  | Parents | 6.3 | 1.3 | 5 | 8 | 6 | 5.3 | 7.5 |
| Deployment of pain  education team | Nurses | 6.7 | 0.9 | 5 | 8 | 7 | 6 | 7 |
|  | Physician | 6.0 | 2.1 | 3 | 9 | 4.5 | 2 | 5 |
|  | Parents | 6.3 | 1.5 | 5 | 8 | 6 | 5 | 7.8 |
| Inflammation of the heel | Nurses | 7.0 | 1.9 | 2 | 9 | 7 | 6 | 8 |
|  | Physician | 5.5 | 1.5 | 3 | 7 | 5.5 | 4.3 | 7 |
|  | Parents | 6.3 | 2.4 | 3 | 8 | 7 | 3.4 | 8 |
| Family physical burden | Nurses | 6.5 | 1.3 | 4 | 9 | 6 | 6 | 7 |
|  | Physician | 6.0 | 1.5 | 4 | 9 | 6 | 5 | 6.8 |
|  | Parents | 6.8 | 1.0 | 6 | 8 | 6.5 | 6 | 7.9 |
| Pneumothorax | Nurses | 6.1 | 1.0 | 5 | 8 | 6 | 5 | 7 |
|  | Physician | 5.9 | 1.2 | 4 | 7 | 6 | 5 | 7 |
|  | Parents | 7.8 | 1.5 | 6 | 9 | 8 | 6.3 | 9 |
| Pain measurement assessment reassessment cycle | Nurses | 6.5 | 1.5 | 3 | 8 | 7 | 6 | 8 |
|  | Physician | 5.8 | 2.1 | 3 | 9 | 6 | 4 | 7 |
|  | Parents | 6.5 | 1.0 | 6 | 8 | 6 | 6 | 7.5 |
| Wound inflammation | Nurses | 7.1 | 0.9 | 5 | 8 | 7 | 7 | 8 |
|  | Physician | 4.8 | 1.5 | 3 | 7 | 4.5 | 3.3 | 6 |
|  | Parents | 6.3 | 2.7 | 3 | 8 | 7 | 3.8 | 8 |
| Sleep | Nurses | 6.9 | 1.4 | 4 | 9 | 7 | 6 | 8 |
|  | Physician | 5.3 | 1.3 | 3 | 7 | 5.5 | 4.3 | 6 |
|  | Parents | 6.3 | 1.9 | 5 | 9 | 5.5 | 5 | 8.3 |
| Postoperative infection | Nurses | 6.7 | 1.1 | 5 | 8 | 7 | 6 | 8 |
|  | Physician | 5.0 | 1.5 | 3 | 7 | 5.5 | 3.3 | 6 |
|  | Parents | 7.3 | 1.5 | 6 | 9 | 7 | 6 | 8.8 |
| Length of respiratory | Nurses | 6.3 | 1.4 | 3 | 8 | 6 | 6 | 7 |
|  | Physician | 5.6 | 1.7 | 3 | 7 | 6.5 | 4 | 7 |
|  | Parents | 7.0 | 1.8 | 5 | 9 | 7 | 5.3 | 8.8 |
| Overdose of analgesics | Nurses | 6.4 | 1.1 | 5 | 8 | 6 | 5 | 7 |
|  | Physician | 5.8 | 1.4 | 3 | 7 | 6 | 5 | 7 |
|  | Parents | 6.5 | 1.0 | 6 | 8 | 6 | 6 | 7.5 |
| Pneumonia | Nurses | 6.0 | 0.9 | 5 | 8 | 6 | 5 | 7 |
|  | Physician | 5.4 | 1.6 | 3 | 7 | 5.5 | 4 | 7 |
|  | Parents | 7.8 | 1.5 | 6 | 9 | 8 | 6.3 | 9 |
| Side effects associated with drug administration | Nurses | 5.8 | 1.2 | 3 | 7 | 6 | 5 | 6 |
|  | Physician | 6.0 | 1.1 | 4 | 7 | 6 | 5.3 | 7 |
|  | Parents | 7.0 | 1.2 | 6 | 8 | 7 | 6 | 8 |
| Duration of blood collection | Nurses | 6.7 | 1.4 | 3 | 8 | 7 | 6 | 8 |
|  | Physician | 5.0 | 1.5 | 3 | 7 | 4.5 | 4 | 6.8 |
|  | Parents | 6.3 | 2.5 | 3 | 9 | 6.5 | 3.8 | 8.5 |
| Frequency of pain  measurement by tool | Nurses | 6.5 | 1.4 | 3 | 8 | 7 | 6 | 7 |
|  | Physician | 5.3 | 2.0 | 3 | 9 | 4.5 | 4 | 6.8 |
|  | Parents | 6.5 | 1.3 | 5 | 8 | 6.5 | 5.3 | 7.8 |
| Heel contusion | Nurses | 6.5 | 1.9 | 2 | 9 | 6 | 6 | 8 |
|  | Physician | 5.3 | 1.3 | 3 | 7 | 5.5 | 4.3 | 6 |
|  | Parents | 5.8 | 2.0 | 3 | 8 | 6 | 3.8 | 7.5 |
| Family length of stay | Nurses | 6.5 | 1.0 | 5 | 8 | 6 | 6 | 7 |
|  | Physician | 5.1 | 1.4 | 3 | 7 | 5.5 | 4 | 6 |
|  | Parents | 6.3 | 0.5 | 6 | 7 | 6 | 6 | 6.8 |
| Suppression of intestinal peristalsis | Nurses | 5.9 | 0.8 | 4 | 7 | 6 | 6 | 6 |
|  | Physician | 5.6 | 1.9 | 3 | 7 | 6.5 | 4 | 7 |
|  | Parents | 6.3 | 1.3 | 5 | 8 | 6 | 6.3 | 7.5 |
| Psychological burden on staff | Nurses | 6.1 | 1.4 | 4 | 9 | 6 | 5 | 7 |
|  | Physician | 5.4 | 1.2 | 4 | 7 | 5 | 4.3 | 6.8 |
|  | Parents | 6.3 | 0.5 | 6 | 7 | 6 | 6 | 6.8 |
| Good mood arousal | Nurses | 6.3 | 1.8 | 2 | 9 | 6 | 6 | 7 |
|  | Physician | 4.8 | 1.4 | 3 | 7 | 5 | 3.3 | 5.8 |
|  | Parents | 6.8 | 1.0 | 6 | 8 | 6.5 | 7 | 7.8 |
| Duration of surgery | Nurses | 6.0 | 1.4 | 3 | 8 | 6 | 5 | 7 |
|  | Physician | 5.5 | 1.4 | 3 | 7 | 6 | 4.3 | 6.8 |
|  | Parents | 6.0 | 2.5 | 3 | 8 | 6.5 | 3.5 | 8 |
| Duration of disease healing | Nurses | 5.9 | 1.6 | 2 | 8 | 6 | 5 | 7 |
|  | Physician | 5.1 | 1.5 | 3 | 7 | 5 | 3.5 | 6.8 |
|  | Parents | 6.5 | 2.6 | 3 | 9 | 7 | 3.8 | 8.9 |
| Physical burden on staff | Nurses | 5.9 | 1.7 | 3 | 9 | 6 | 5 | 7 |
|  | Physician | 5.3 | 1.3 | 4 | 7 | 5 | 4 | 6.8 |
|  | Parents | 6.3 | 0.5 | 6 | 7 | 6 | 6 | 6.8 |
| Methemoglobinemia | Nurses | 5.5 | 0.9 | 3 | 6 | 6 | 5 | 6 |
|  | Physician | 5.5 | 1.2 | 3 | 7 | 6 | 5 | 6 |
|  | Parents | 6.5 | 1.7 | 5 | 8 | 6.5 | 5 | 8 |
| Duration of oxygen  administration  during ventilation | Nurses | 5.9 | 1.2 | 3 | 8 | 6 | 6 | 6 |
|  | Physician | 4.8 | 1.5 | 2 | 6 | 5 | 4 | 6 |
|  | Parents | 6.8 | 1.7 | 5 | 9 | 6.5 | 5.3 | 8.5 |
| Length of hospital stay | Nurses | 6.1 | 1.3 | 3 | 7 | 7 | 5 | 7 |
|  | Physician | 5.0 | 1.6 | 3 | 7 | 5 | 3.3 | 6.8 |
|  | Parents | 5.8 | 1.3 | 4 | 7 | 6 | 4.5 | 6.8 |
| Constipation | Nurses | 5.6 | 1.0 | 3 | 7 | 6 | 5 | 6 |
|  | Physician | 5.1 | 1.5 | 3 | 7 | 5 | 4 | 6.8 |
|  | Parents | 6.5 | 2.7 | 3 | 9 | 7 | 3.8 | 8.8 |
| Length of time to establish  tube feeding | Nurses | 5.5 | 1.0 | 3 | 7 | 6 | 5 | 6 |
|  | Physician | 5.6 | 1.4 | 3 | 7 | 6 | 4.5 | 6.8 |
|  | Parents | 5.8 | 2.1 | 3 | 8 | 6 | 3.8 | 7.5 |
| Physical growth | Nurses | 6.0 | 0.8 | 5 | 8 | 6 | 6 | 6 |
|  | Physician | 4.8 | 2.6 | 2 | 9 | 5 | 2 | 6.8 |
|  | Parents | 6.0 | 1.2 | 5 | 7 | 6 | 5 | 7 |
| Out-of-pocket expenses  not covered by medical  insurance | Nurses | 5.8 | 1.2 | 3 | 7 | 6 | 5 | 7 |
|  | Physician | 4.8 | 1.6 | 3 | 7 | 5 | 3 | 6 |
|  | Parents | 6.0 | 0.8 | 5 | 7 | 6 | 5.3 | 6.8 |
| Vomiting | Nurses | 5.5 | 1.2 | 3 | 7 | 6 | 5 | 6 |
|  | Physician | 4.9 | 1.4 | 3 | 7 | 5 | 3.5 | 5.8 |
|  | Parents | 6.3 | 1.7 | 4 | 8 | 6.5 | 4.5 | 7.8 |
| Hyperglycemia | Nurses | 5.5 | 1.4 | 3 | 8 | 6 | 5 | 6 |
|  | Physician | 5.0 | 1.5 | 3 | 7 | 5.5 | 3.3 | 6 |
|  | Parents | 6.0 | 1.9 | 4 | 8 | 6 | 4.3 | 7.8 |
| Body weight | Nurses | 6.0 | 1.3 | 3 | 8 | 6 | 6 | 7 |
|  | Physician | 4.3 | 1.8 | 1 | 6 | 4.5 | 3 | 6 |
|  | Parents | 6.0 | 0.8 | 5 | 7 | 6 | 5.3 | 6.8 |
| Nausea | Nurses | 5.5 | 1.2 | 3 | 7 | 6 | 5 | 6 |
|  | Physician | 4.4 | 1.1 | 3 | 6 | 4.5 | 3.3 | 5 |
|  | Parents | 6.0 | 1.4 | 4 | 7 | 6.5 | 4.5 | 7 |
| Swallowing | Nurses | 5.4 | 0.9 | 3 | 6 | 6 | 5 | 6 |
|  | Physician | 4.5 | 1.2 | 3 | 6 | 4.5 | 3.3 | 5.8 |
|  | Parents | 6.0 | 1.4 | 4 | 7 | 6.5 | 4.5 | 7 |
| Expenses covered by medical insurance | Nurses | 5.4 | 1.6 | 3 | 8 | 6 | 4 | 6 |
|  | Physician | 4.5 | 1.7 | 2 | 6 | 5 | 3 | 6 |
|  | Parents | 5.8 | 1.0 | 5 | 7 | 5.5 | 5 | 6.8 |
| Head circumference | Nurses | 5.7 | 1.0 | 4 | 8 | 6 | 5 | 6 |
|  | Physician | 3.9 | 1.6 | 2 | 6 | 4.5 | 2 | 5 |
|  | Parents | 5.3 | 1.3 | 4 | 7 | 5 | 4.3 | 6.5 |
| Height | Nurses | 5.2 | 1.0 | 3 | 7 | 5 | 5 | 6 |
|  | Physician | 3.9 | 1.6 | 2 | 6 | 4.5 | 2 | 5 |
|  | Parents | 5.0 | 1.6 | 3 | 7 | 5 | 3.5 | 6.5 |
| Skin rash | Nurses | 4.8 | 1.3 | 3 | 6 | 5 | 3 | 6 |
|  | Physician | 3.6 | 0.9 | 3 | 5 | 3 | 3 | 4.8 |
|  | Parents | 5.3 | 1.7 | 3 | 7 | 5.5 | 3.5 | 6.8 |

Note: Nurse includes evaluation of nurse practitioner and physical therapist; Nurses (n=11), Physician (n=8), Parents (n=4). Outcomes are ranked as overall mean scores.
